# Language Model Applications to Spelling with Brain-Computer Interfaces

**DOI:** 10.3390/s140405967

**Published:** 2014-03-26

**Authors:** Anderson Mora-Cortes, Nikolay V. Manyakov, Nikolay Chumerin, Marc M. Van Hulle

**Affiliations:** Laboratorium voor Neuro- en Psychofysiologie, KU Leuven, Campus Gasthuisberg, O&N2, Herestraat 49, Leuven B-3000, Belgium; E-Mails: Nikolay.Manyakov@gmail.com (N.V.M.); Nikolay.Chumerin@med.kuleuven.be (N.C.); Marc.VanHulle@med.kuleuven.be (M.M.V.H.)

**Keywords:** Ambient Assisted Living, Brain-Computer Interfaces, spelling systems, electroencephalography, communication systems, language models

## Abstract

Within the Ambient Assisted Living (AAL) community, Brain-Computer Interfaces (BCIs) have raised great hopes as they provide alternative communication means for persons with disabilities bypassing the need for speech and other motor activities. Although significant advancements have been realized in the last decade, applications of language models (e.g., word prediction, completion) have only recently started to appear in BCI systems. The main goal of this article is to review the language model applications that supplement non-invasive BCI-based communication systems by discussing their potential and limitations, and to discern future trends. First, a brief overview of the most prominent BCI spelling systems is given, followed by an in-depth discussion of the language models applied to them. These language models are classified according to their functionality in the context of BCI-based spelling: the static/dynamic nature of the user interface, the use of error correction and predictive spelling, and the potential to improve their classification performance by using language models. To conclude, the review offers an overview of the advantages and challenges when implementing language models in BCI-based communication systems when implemented in conjunction with other AAL technologies.

## 1. Introduction

**Figure 1.**
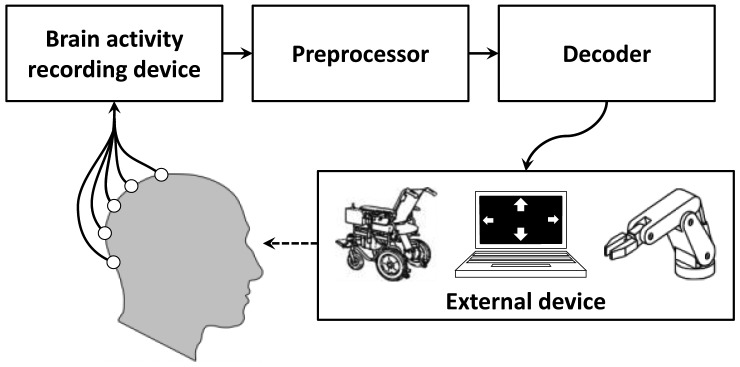
Brain-Computer Interface scheme.

A Brain Computer Interface (BCI) enables a user to communicate with the external world by directly translating his/her brain activity into (computer) commands without relying on the brain's normal output pathways. Due to this, BCIs have raised great hopes in providing alternative communication means for persons suffering from motor disabilities such as amyotrophic lateral sclerosis (ALS), spinal cord injuries or brain paralysis [1–3], and other users targeted by the Ambient Assisted Living (AAL) community [4], provided their sensory and cognitive functions are still intact [5]. Since one of AAL's aims is to improve the quality of life of elderly persons with disabilities, BCI systems have become an opportunity to achieve this via different AAL implementations of BCI systems [4,6–8]. A BCI system in general (see Figure 1) normally comprises the following components: (i) a device that records the brain activity which is either invasive (e.g., electrocorticography) or non-invasive (e.g., electroencephalogram (EEG)); (ii) a *preprocessor* that reduces noise and artifacts, prepares the signals for further processing and extracts the relevant information from the recordings; (iii) a *decoder* that classifies the extracted relevant information into a control signal for (iv) an *external device* that could be any type BCI-compatible application (e.g., a robotic actuator, a prosthesis, a computer screen *etc*.), and that provides feedback to the user. This *external device* could also be used for evoking brain activity, thus serving as a *stimulation unit* (see Section 2 for examples). The feedback to the user is an important aspect of the BCI system as it provides the former with information about mistakes (by the decoder or the user) and in this way motivates the user to better modulate his/her brain activity and to increase attention and engagement in the task, thus adhering to a so-called *neurofeedback* principle. As a result, the BCI can be regarded as a control system with active feedback (closed-loop system).

The first BCI was presented in the pioneering work of Vidal [9], where the basic requirements of a man-machine communication tool and the concepts, feasibility, possibilities and even its limitations were already introduced. Since then BCI applications have ramified into different areas such as clinical/translational research (from basic research to clinical BCI implementations) [10], entertainment [11], ambient assisted living [6], and emerging applications such as bionic assistive devices [12], and the detection of covert behavior, among others (see [13] for a review).

Since invasive BCI requires surgery and faces not only ethical but also technical challenges, it has rarely been performed on on humans [14]. It therefore comes as no surprise that the non-invasive alternative became widely adopted in human BCI-based communication research. Among all noninvasive BCIs, the EEG-based ones are favored above other non-invasive ones such as functional magnetic resonance imaging (fMRI) [15], magnetoencephalography (MEG) [16] and functional near infrared spectroscopy (fNIRS) [17,18]. The advantages of EEG led to a rapid increase in the number of BCI research groups all over the world [19] as reflected by the share in the number of publications in the field in the last decade [20,21], and have spurred the interest in developing feasible and practical BCI systems, as covered in several review papers [3,21–25], and some of which have been implemented within AAL applications as control environment [6] and social interaction [4]. More specific reviews focus on communication issues [13,22,26–28], signals related issues such as their processing [29–31], feature extraction [18,32], brain potentials [33], neuroimaging-based BCI [34], handling artifacts [31] and decoding methods for BCI systems [35,36]. Nonetheless, to the best of the authors' knowledge, there is no comprehensive review of the applications of language models in BCI systems despite of the increasing research interest in this direction (see [3,25,29,37] for more detailed reviews of BCI systems).

The aim of this article is to review the available literature that combines language models with BCI systems for communication applications. Since research in this direction has been performed only for EEG-based BCI, we also limit ourselves to this case. Nevertheless, all language modeling strategies discussed below could in principle also be used in other BCI types, including different AAL applications (e.g., controlling environment). The focus of this paper is on BCI spellers (which are systems allowing users to type individual characters, words or even sentences by decoding their brain activity) combined with applications such as word prediction, completion, error correction, and so on, which may increase the communication speed without increasing the user's cognitive load. These new approaches offer a significant advantage over other augmentative and alternative communication (AAC) devices, which at least require some degree of motor activity [38,39].

## 2. Paradigms for BCI Communication Systems

One of the main applications for BCI is spelling. These spelling systems are mainly based on one of three BCI paradigms, exploiting different types of brain responses: *event-related potentials* (ERP), *steady state evoked potential* (SSVEP) or *frequency visual evoked potential* (f-VEP) and *motor imagery* (MI).

### 2.1. ERP-Based BCI

**Figure 2.**
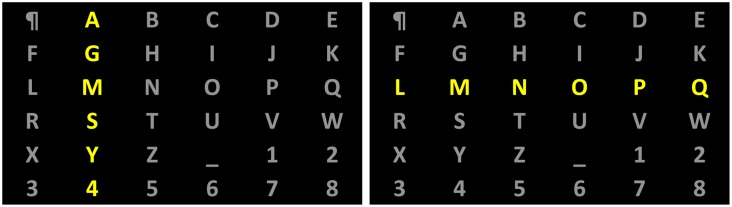
Example of typing matrix in P300-speller. Rows and columns are intensified in random order. The intensification of the second column (**Left panel**) and the third row (**Right panel**) are shown. One round consists of the intensification of each one of the six columns and six rows.

The most known representative of this group is the so-called P300-speller. The idea behind it derives from the observation that a stereotypical component of brain potential is evoked in response to an infrequent stimulus attended by the user, while it is absent for a frequent but non-attended stimulus. The main difference in responses is seen in a positive deflection around 300ms following onset of the stimulus, the so called P300 component, which is primary generated above the parietal and central cortices [40]. This phenomenon is present for visual [1], auditory [41] or tactile stimulations [42,43], which led to different BCI interaction modes. A first speller of this type was a visual one, proposed in [40]. In such visual P300-spellers a 6 × 6 matrix with characters is displayed with rows and columns intensified in random order (see Figure 2) with about 5–6 intensifications per second [40,44]. The user attends to one of the symbols he/she wishes to communicate. The intensification of the row/column that contains the desired character evokes an enhanced P300 component [40]. The trained (in advance) classifier detects the row-column combination for which the P300 response is present and selects the character accordingly. The recorded signal is a superposition of the activity related to the stimulus and all other ongoing brain activity together with noise, which makes single-trial ERP detection very difficult. In order to more robustly detect ERPs, recordings over several row/column intensification rounds need to be averaged. By averaging the activity that is time locked to a known event (e.g., the onset of the attended stimulus) is extracted as an ERP, whereas the activity that is not related to the stimulus onset is expected to be averaged out. The speed with which characters can be typed therefore heavily depends on the number of rounds needed to extract the P300 component accurately. Although such BCIs are mainly regarded as P300-based, other components of evoked potentials also play important role in decoding [45].

BCIs based on *rapid serial visual presentation* (RSVP) [46–49] could also be categorized as ERP-based BCI. RSVP-based BCI uses visual stimuli presented with a rate of about 10 stimuli per second [46–48], among which user attends to the only stimulus he/she wish to communicate. Stimuli are rapidly displayed in a one-by-one basis in the same predefined position known to the user in order to avoid necessity for their search and eye movements, as they could produce artifacts in the EEG recordings. The user has to attend the desired stimulus and mentally count the number of times it is presented. The decoding procedure is similar to the P300-based case.

BCI based on *motion-onset* [50–53] and *transient visual evoked potentials* (t-VEP) BCI [54] also fall in this BCI category and utilize quite similar processing and decoding techniques. Motion-onset VEP is evoked by the presentation of motion stimuli [55], and its main components have been described as P100, N200 and P200 [56]. The t-VEPs are the responses recorded from the visual cortex after a visual stimulus has been displayed [57] and the amplitude of the visual response increases every time the target is closer to the subject's central visual field [58].

Much research has been directed towards achieving a higher detection accuracy of brain evoked responses to target stimuli for an equal or lower number of intensification rounds. This research was primary performed in the *preprocessing component* (see Figure 2), searching for a better spatial and frequency filtering or a better feature selection and construction methods [59–62], on the *classifier component* [63], and in the design of the *external-stimulation device*, e.g., by adapting the inter-stimulus interval [40], the size of the matrix [1] and the intensification protocol [64–66].

ERP-based BCIs are also known by the fact, that those systems do not necessary depend on the gaze direction, *i.e.*, they could rely on covert attention instead [41,49,67].

### 2.2. BCIs based on Frequency and Code Modulation of VEP

**Figure 3.**
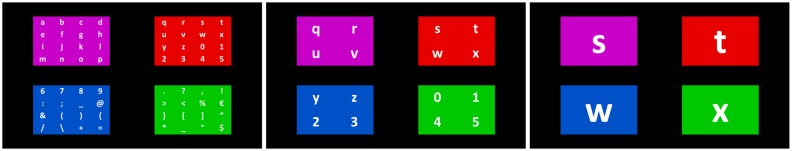
Three consecutive stages to select symbol “w” in a SSVEP speller.

The *steady-state visual evoked potential* (SSVEP) or *frequency visual evoked potential* (f-VEP) [68], recorded above the occipital cortex, is the response to a periodic presentation of a visual stimulus (*i.e.*, a flickering stimulus). When the stimulus is flickering at a sufficiently high rate (starting from 6 Hz), the individual evoked responses to each stimulus flash will overlap, leading to a steady-state signal resonating at the stimulus flicker rate and its integer multipliers (harmonics) [69]. With such a paradigm it is possible to detect whether a subject is looking at a stimulus flickering at frequency *f*, by verifying the saliency of the frequency *f* and possibly also its harmonics, *2f*, *3f*, … in the spectrum of the recorded EEG signal. Similarly, one can detect which stimulus, out of several of them (each one flickering at a different frequency), is gazed at by the subject, by checking the corresponding frequencies and their harmonics. Linking each flickering stimulus to a particular command, a multi-command frequency-coded SSVEP-based BCI can be implemented. For example, one can construct a speller by dividing the screen into quadrants, flickering at different frequencies, which contain different subsets of characters (Figure 3). The user gazes at the quadrant that contains the desired character [70], allowing the selection of any character (here out of 64) by performing consecutive quadrant selections (three for Figure 3).

Since in the spectral domain the EEG amplitude decreases as the frequency increases, the higher stimulus frequencies and harmonics become less prominent. Furthermore, the SSVEP is embedded in other ongoing brain activity and (recording) noise [70]. Thus, when considering a recording interval that is too small to reliably extract the frequency components, erroneous detections are quite likely to occur. To overcome this problem, averaging over several recording intervals [71], or recording over longer time intervals [58] are often used together with a spatial filtering strategy [72–74] to increase the signal-to-noise ratio (SNR). An efficient SSVEP-based BCI speller should be able to reliably detect several frequencies, which makes the detection issue even more complex, calling for efficient signal processing and decoding algorithms. This has primary led to modifications in the preprocessing and classifier components of Figure 1.

An additional limitation arises from the stimulation side: only stimulation frequencies within a particular frequency range evoke a reasonable SSVEP response [75]; the harmonics of some stimulation frequencies could interfere with one another, leading to a deterioration of the decoding performance [76], even more so when the stimulation frequencies depends on the refresh rate of the screen [76] (in the case of stimulation on a computer screen). This encouraged the search for alternative stimulation methods in computer screen based SSVEP BCIs [77] or other encoding methods [75,78,79], thus, modifying the stimulation (external) device block in Figure 1.

Another VEP-based technique adopted by BCIs is called *code modulated VEP* (c-VEP) originally proposed by Sutter [80] and further developed by others [68,81,82]. Following c-VEP approach to induce most distinguishable visual responses to different target stimuli, the intensity of the stimuli is modulated by a special pseudorandom binary sequence, so-called m-sequence, which is designed to be nearly orthogonal with respect to its shifted versions. This m-sequence and its (circularly) shifted versions are then used to modulate visual stimulation to induce discernible brain responses. The processing of the c-VEP responses involves averaging across several epochs, where each epoch corresponds to one full presentation of the m-sequence during stimulation. The decoding step usually relies on simple template matching technique: the preprocessed (filtered and averaged) c-VEP response is matched against several pre-computed templates, corresponding to the target stimuli, and the winner is selected as the best matching one. Some other classification methods (e.g., one class SVM, canonical correlation analysis) have been proposed [82] to improve the performance of c-VEP BCIs.

More detailed descriptions of c-VEP techniques as well as RSVP, motion-onset, and t-VEP techniques can be found in [46,50,54,68,83].

### 2.3. MI-Based BCI

A *motor imagery* (MI) BCI is based on changes in neural populations in the motor cortex when performing an actual or imagined limb movement. These changes are hypothesized to be due to decrease (event-related desynchronization, ERD) or an increase (event-related synchronization, ERS) in the synchrony of the underlying neuronal populations [84,85]. In spectra of EEG, recorded above motor cortex contralaterally to moved (or imagined to be moved) body part (e.g., left arm), this (imaginary) movement produces a decrease in power (ERD) in the mu (8–13 Hz) and beta (13–26 Hz) band in comparison to the absence of such movement or its imagination [86]. As such, by determining changes in spectral amplitudes in the corresponding frequency bands or, equivalently, in the variance of the EEG signal filtered in the same band one can determine the subject's intentions [87]. In addition to ERD/ERS, the readiness potential (Bereitschaftpotential) has also been used for the decoding of motor imagery [88,89]. By involving two or more different limbs, for example, the right and left hands, and relying on the fact that different parts of the motor cortex are responsible for different limbs (*i.e.*, they are spatially distributed), one can build a BCI system. In order to enhance the detectability of the MI, different fixed- [90], data-driven [91–93], multi-class [94] spatial filtering approaches have been proposed, as well as different classifiers [95], thus mainly modifying the *Preprocessor* and *Decoder* blocks of Figure 1. A detailed review of spatial filtering and classification techniques for MI-based BCIs can be found in [96,97].

Several MI-based spelling devices have been proposed. For example, D'Albis in [98] used a typing interface consisting of 26 characters of the English alphabet and a “space” (thus, 27 symbols in total) equally divided into three groups (see Figure 7b). The user selects one of those three groups or the “undo” command by imagining the movement of the corresponding body part(s) (in their case the right hand, left hand, both hands or both feet). By selecting one of these groups, the nine characters are divided into three new groups of three characters. And so on. Thus, typing one character takes three consecutive selections, similar to the SSVEP speller described above in Section 2.2.

**Figure 4.**
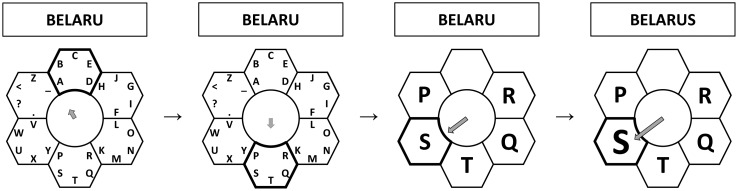
Two different types of imaginary movements allow the user to control the rotation and extension of the gray arrow in the Hex-o-Spell interface. In this example the last letter in the word “BELARUS” is selected. Adapted from [99].

Another MI-based BCI-speller is the so-called Hex-o-Spell [99], with which 30 different characters can be typed by imagining one of two movements (right hand and foot) (see Figure 4). The characters are shown in six adjacent hexagons distributed around a circle. Each hexagon contains five characters and a “go back” command. For the selection of the hexagons, there is an arrow in the center of the circle. Right hand movement imagination controls the rotation of the arrow clockwise. The imagination of the foot movement extends the arrow until it reaches one of the hexagons after which it is selected. After this, the characters in all hexagons, except for the selected one disappear, while the remaining characters and the “go back” command are mapped into six hexagons around the circle, *i.e.*, the same layout as in the beginning. Using the same arrow-based strategy, the user selects the desired character or decides to go back to the previous level of the interface to correct a mistake.

## 3. Language Model in BCI Spellers

As discussed before, the conventional approach to improve the communication speed and accuracy of a BCI speller is to search for new and better signal processing and classification algorithms, or to change the stimulation mode or stimulation parameters, thus, modifying the blocks in Figure 1. Albeit successful to some extent, BCI spellers still cannot compete with their assistive technology counterparts. This prompts for alternative solutions beyond the ones covered by traditional BCI research. One such solution was indicated by Donchin and coworkers in [100]: “*It is well known that there are substantial sequential dependencies in English. It is our intent to incorporate information about the sequential structure of the language in the next phase of the development of the BCI. Similarly, it is possible to incorporate spelling correction software so that spelling mistakes can be managed even as increases in the operational speed may be associated with increased number of errors*.” While this was proposed already in 2000 and seemed quite promising, until recently there were no attempts to adopt language model strategies. In the following subsections, we describe recent developments and implementation of language models for BCI spellers.

### 3.1. Language-Driven Design of Static User Interfaces

As a basic implementation of language models in BCI spellers, one can mention the way characters are arranged in the spelling interface. The characters' layout could rely on the initial probability of occurrence of a character in a particular language or on the aim to minimize typing mistakes with respect to some dictionary. Such a layout is fixed and does not change during typing (whence “static”). The corresponding interfaces are dependent on the BCI paradigm adopted.

**Figure 5.**
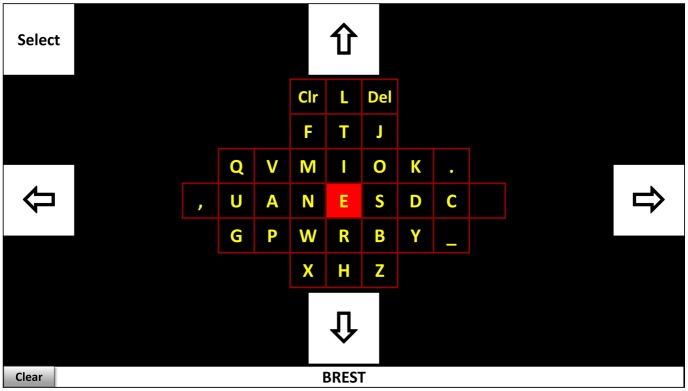
Layout of the Bremen SSVEP-based BCI-speller. Adapted from [101].

An example that accounts for the relative frequency of character occurrence in a language, consider the interface of the Bremen SSVEP-based BCI-speller [101]. It has in the middle of the screen a virtual keyboard with 32 symbols (see Figure 5) surrounded by five boxes flickering at different frequencies. These boxes correspond to commands for navigating the cursor (indicated by red color) “left”, “right”, “up”, “down”, and for selecting the intended character. The application starts with a cursor in the central position corresponding to the most frequent character in English (“E”, in Figure 5). By gazing at the command boxes, the user can navigate the cursor to the desired letter and confirm his/her choice with the “select” command. The further the character is located from the center, the more command selections (cursor movements) are required. Letters with the higher frequency of occurrence are positioned closer to the center while the less frequent ones are further away.

Moreover, attentional switches are also taken into account. For example, two commands (left-left) are required to reach the letter “A” and the same amount of commands (left-up) to reach the letter “M”. But in the latter case the user has to redirect his/her gaze from the command box “left” into command box “up”, while in the former case such a redirection of the gaze is not required, which is more easy for the user. Considering this, the more frequent letter “A” (8.167% according to [102]) is positioned in a more easily reachable position than the less frequent letter “M” (2.406% according to [102]). By accounting for the initial letter probability in a language the user can more easily and much faster select the intended characters with this interface, which in turn results in a higher throughput of the system.

**Figure 6.**
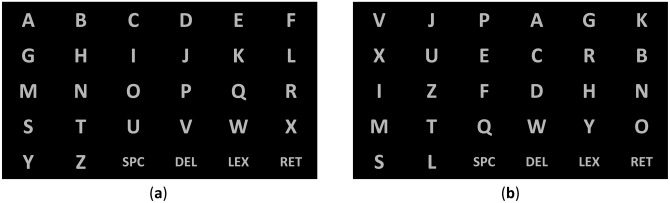
Conventional (**a**) and modified (**b**) P300-speller interfaces used in the study of Ahi and colleagues [103]. Adapted from [103].

Another way to place characters in static interface, but this time for a P300 row-column speller, was proposed in [103]. The authors tried to modify the spelling matrix by taking into account the notion that the majority of errors in a row-column paradigm occur either by wrongly selecting a row or a column [63]. The idea was to displace as much as possible letters which are different in similar words proved by a dictionary attached to the speller. For example, the words HINT and HUNT are similar, since they differ only in the second letter. While using the conventional interface of Figure 6a, one can see that letters “I” and “U” are in the same column. In the modified interface of Figure 6b neither the row, nor the column for the letters “I” and “U” coincide. In this way, with a conventional interface, while typing the word HINT and making mistakes in the second letter, one could end up in the wrong word HUNT, even when the column is identified correctly (but not the row), but in the case of the modified interface, a row or column misclassification during the selection of the second letter in the word HINT will not lead to a conventional English word. This could be an indication that a decoding mistake was made, which could be rectified, e.g., by using the algorithm described in Section 3.5.

### 3.2. Dynamic Adaptation of User Interface

The user interfaces discussed in Section 3.1 are static ones, *i.e.*, they do not change during the spelling process. However, it is known that the probability of a letter in a word depends on the previously typed ones. For example, if one has already typed ENGL, it is not likely to have X as the next letter , while it is quite probable to have I as the next letter (for example, the word ENGLISH). Thus, the probability of each letter in a language is not fixed *a priori*, but varies during spelling. This idea was employed in the group of methods described below, all of which perform dynamic adaptations of the user interface, depending on the already (partially) spelled text.

**Figure 7.**
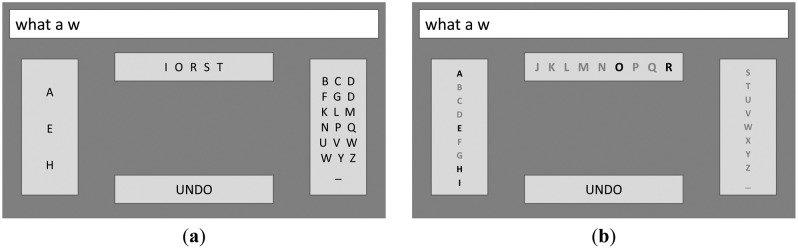
Dynamic (**a**) and static (**b**) interfaces for a system, that considers previously typed text “what a w” for spelling the next letter. Adapted from [98].

In addition to their standard interface (see Section 2.3 for a description), D'Albis [98] also foresaw dynamic modifications by incorporating a language model for taking into account the changing probability of the next letter *l_n_* in the currently typed word, based on the already typed characters of the same word *l*_1_,…, *l_n_*_−1_ ( prefixes) and the two typed preceding words *w*_1_ and *w*_2_. These modifications are based on an algorithm that extracts from the corpus (*corpus* is a large set of texts used for linguistic analysis), attached to the speller, all possible distinct triples of words, where the first two words are *w*_1_ and *w*_2_, and the first *n* – 1 letters in the third word are *l*_1_,…, *l_n−_*_1_. When estimating the probability of having the next letter *l_n_*, the number of selected triples goes to the denominator while the numerator is equal to the number of triples, among the selected ones, where additionally the *n*-th letter in the third word is *l_n_*. As an example, let us assume one wants to type the phrase “what a wonderful day”, and the two first words “WHAT” (*w*_1_) and “A” (*w*_2_) were already typed. In the third word, the first letter “W” (*l*_1_) was also typed, and the user intends to type the second letter (*l*_2_) (see Figure 7). The algorithm scans the corpus in order to find all triples following “WHAT A W…” (where the dots represent any further characters within the third word, starting with the letter “W”) and estimates the number *N* of such triples. Among the found ones, the algorithm also estimates the number of those that take the form of “WHAT A WA…” (for *l*_2_ = “A”), “WHAT A WB…” (for *l*_2_ = “B”), and so on. By dividing these numbers by *N*, the algorithm generates an estimate of the probability for any letter *l*_2_ to be the next one. All letters with nonzero probability were enabled in the interface (see [98] for further explanation). In the example in Figure 7, after typing “WHAT A W” the algorithm detected a nonzero probability only for the letters “A”, “E”, “H”, “I”, “O”, “R” (ranked by their estimated probabilities), which are considered as the only likely choices for the next character.

The dynamic interface (Figure 7a) then rearranges the candidate characters in descending probability (where the first three most probable letters are grouped together, and so on) in order to minimize the number of subsequent group expansions. The static interface (see Figure 7b) does not change the character layout, but instead disables the ones with zero estimated probability. This interface could be regarded as more comfortable for the subject, since it does not require attentional shifts. Both interfaces enables the user to pick the next letter “O” just by two selections (instead of three in the normal mode), thus making spelling faster.

**Figure 8.**
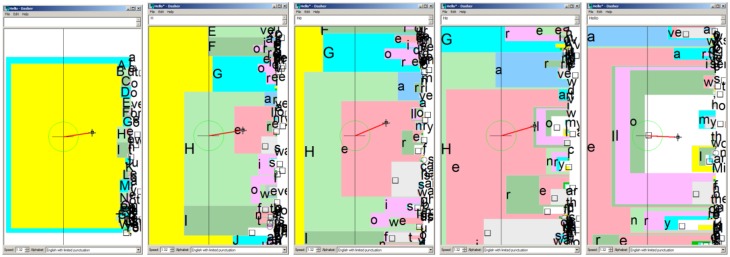
Five successive stages when entering “Hello” with the Dasher interface.

Another strategy that accounts for the previously spelled characters in BCI speller can be based on the Dasher interface [104], which originally employed 2D control. When the pointer (black arrow) is at the center of the screen (indicated by the crosshair), nothing is happening. As soon as the user moves the pointer to the right, the letters on the right hand side of the screen start to zoom in (see Figure 8, showing consecutive screenshots while typing the string “Hello”). The vertical position of the pointer controls the direction in which zooming is performed and the pointer's horizontal position controls the speed of zooming. If one moves the pointer back to the center of the screen, the spelling process pauses, when moving the pointer to the left side of the screen mistakes can be corrected by moving back in the already typed sequence.

The Dasher interface shows symbols that are more probable in the current context by enlarging the square region around them. In the initial stage, the probabilities (size of the squares) of each symbol are taken from the frequencies of each symbol in an adjusted corpus. This makes the Dasher interface different from the one from [98], since it additionally incorporates the strategy discussed in Section3.1. Probabilities of consecutive symbols are estimated with the use of an *n*-gram (*n-gram* is an adjacent sequence of *n* item from a whole sequence.) language model on character's basis; by assessing from the attached corpus and based on previously typed text the probabilities *c*_1_*c*_2_…*c_n_*_−1_*c*, where *c* is the next symbol to be typed, and *c_i_* are the previously typed symbols. An additional difference with the approach from [98] is also the fact that all symbols (not only letters) are considered, *i.e.*, “space”, punctuations and other symbols are assumed to be *c_i_*'s. In this way, the sequence *c*_1_*c*_2_…*c_n_*_−1_*c* could also have symbols not only from the word currently being typed (as in [98]), but also from the ones that are part of the preceding word(s), and spaces and other punctuation symbols between those words.

The idea of using a Dasher interface in a BCI-speller was first mentioned by Wills and MacKay [105] in 2006, but no real BCI application was presented in the paper. Nevertheless, in their paper they acknowledged a potential problem with the inferior 2D control in BCIs, and discussed ways to perform 1D control instead. They suggested either using a special mapping of 1D input into 2D, as required in Dasher, or to fix the zooming speed and allow for only vertical control with the BCI interface. With the latter strategy, one can divide the Dasher interface into several vertically distributed zone-stimuli (as for the case of SSVEP or P300 BCI), and when one of those zones is selected, the pointer will move into the corresponding region for zooming [105]. In real on-line typing, Dasher was evaluated when using motor imagery BCI and the 1D to 2D mapping strategy [39], and when using SSVEP-based BCI (with constant horizontal speed) constructed around only one flickering stimulus for vertical control, where gazing at the stimulus is associated with moving upwards, while no gazing leads to moving downwards [106].

The approaches mentioned so far in this subsection are based on a probability assessment of the next symbol by aggregating statistics from the attached corpus. As an alternative approach [107], one can try to exclude any statistical information and construct in advance a *trie* (*trie*, derived from re*trie*val, is an ordered tree data structure used mainly for managing strings in memory) lexicon structure from the corpus. Mathis and Spohr [107], using all words from the corpus, constructed the trie, where starting from the root node (associated with an empty string) and by moving down to descendant nodes and further on, one can “read” all words from this corpus. When constructed in this way, a trie is another representation of all words from a corpus. When used in a BCI-speller, when the user is typing, any entered string is monitored and associated with the corresponding node in the trie. If the current node has only a single edge exiting from it, the corresponding next character (associated with this edge) is incrementally added to the already typed text. Thus, such a strategy allows adding a uniquely determined next character, speeding up the text spelling process. For example, if one wants to type the word UNIQUE, after spelling UNIQ the next letter “U” will be added automatically, since it is the only possible continuation of the previously typed sequence in English. Mathis and Spohr in [107] used this strategy in a simulated P300 speller and found that, on average, every eighth character was added automatically, allowing to speed up the typing process, while retaining a very low rate (0.84%) of wrong word completions.

### 3.3. Minimization of Command Selections by Using T9-Like Interface

T9, which stands for Text on 9 keys [108], is a language interface developed by Tegic Communications [109] for text entering on mobile phones. This system was designed to enable typing of more than 30 different characters with only numerical keys on a mobile phone's keypad. Each key corresponds to several characters. For example, if one wants to type HOME then, with the T9 interface, where key “4” corresponds to “G”, “H”, “I”, key “6” to “M”, “N”, “O” and key “3” to “D”, “E”, “F”, he/she needs to select keys 4663. After this, T9 looks through an attached dictionary in order to find all words corresponding to this sequence of key presses, and ranks them by their frequency of use. For example, 4663 corresponds to HOME, GOOD, GONE… The most frequent words are presented to user for selection (the exact number of those words depends on interface). The T9 system modifies the word frequencies depending on the user, by increasing word frequencies according to the history of typing, and also allows for typing new words that are subsequently added to the dictionary. Thus, the T9 interface minimizes the number of key strokes, which is a big advantage for BCI-spellers with limited number of commands to select from (*i.e.*, stimuli). While the system initially was called T9, it actually uses 12 keys: keys 2–9 for letters, other keys for punctuation, space and other characters.

Höhne and co-workers in [41] used the T9 system for an auditory ERP-based speller, where a 3 × 3 spelling matrix was encoded by three levels of sound pitches (high, medium and low) for the rows and three directions of sound (left, middle and right) for the columns. They changed the original T9 interface in order to use only nine keys instead of 12. In spelling mode, keys 2–9 were connected to letters, as in an ordinary T9, but key 1 was for switching the interface to a mode in which keys 4–8 encode five most frequent words suggested by T9, and keys 1–3 and 9 correspond to punctuation, backspace, delete and exit, respectively.

A similar system was also implemented in the visual P300 Chinese speller [110]. In this system, each symbol can be spelled with five strokes used for writing any Chinese symbol. After typing the intended sequence of strokes, the seven most frequent Chinese words were presented to the user for selection.

### 3.4. Predictive Spelling Module in BCI Spellers

This approach is based on the psycholinguistic cohort model proposed in [111]. The model states that when a person hears or reads a segment (consisting of several consecutive letters) of a word, all words from his/her lexicon starting from this segment are “activated” in his/her brain. The more letters are added to the segment, the fewer words remain “activated”. Thus, by adding more and more letters to the segment, the “activation” is narrowing down to only one word, *i.e.*, the one that coincides with the word being read or heard.

Such a psycholinguistic model could be used for a spelling interface when the interface is connected to some dictionary or corpus (*i.e.*, the user's lexicon is replaced by words from this dictionary or corpus). When the user has typed the first letters of the intended word, all words from the attached dictionary that share the same first letters are “activated”, and the most frequent words among them are presented to a target list from which the user can select. The user then can either further type the intended word letter-by-letter with the BCI speller, or select the intended word as soon it appears in the list. In this way, one expects the user to be able to spell faster, since not always the whole word needs to be typed character-by-character.

Depending on the interface, the word suggestion list could be presented either on separate layout, than the one for character-by-character spelling [112,113], which requires an additional BCI command to switch between those two layouts, or it could be incorporated into the ordinary layout, thus not requiring any additional switches, which saves time [114,115].

**Figure 9.**
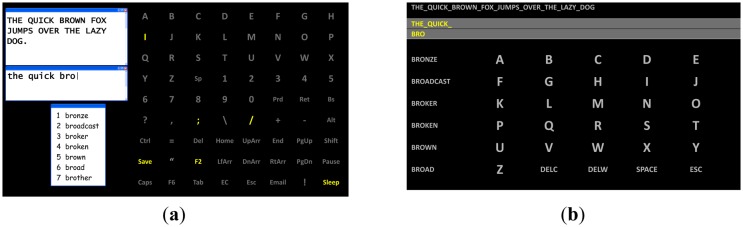
Two different layouts designed for predictive spelling. (**a**) The predicted words are displayed on the left side of the screen over an “extra” window in the interface, thus requiring keeping them in the user's memory, which could increase the cognitive workload. Adapted from [114]. (**b**) The solution proposed to alleviate the cognitive workload by integrating the suggested words into the interface as selectable stimuli. Adapted from [115].

Similar to other alternative and augmentative communication (AAC) devices [116,117], a BCI predictive spelling may increase the user's cognitive workload [113,114]. This was observed in P300 spellers, where a list of suggested words was displayed, but they were not used directly as a stimuli for selection, but the words were labeled by numbers 1–7, and the subject had to type the corresponding number in order to select one of those words (see Figure 9a) [114]. By modifying the interface, so that words from the list are integrated directly into interface, thus they are used as the stimuli, the above mentioned problem could be alleviated (see Figure 9b) [115].

When word suggestions are shown to the user, they are visualized by presenting only a few of the most likely ones based on the system's lexicon. Those frequencies could initially be equal for all words in the lexicon and change according to the typed text [112,114], or they could be different and depend on word frequency, derived from the corpus used for compiling the lexicon [115]. Moreover, the word frequencies could be estimated for each word separately [115], *i.e.*, not taking into account the context, or by also accounting for one or more preceding words [98], *i.e.*, an *n*-gram model on words basis.

### 3.5. Spelling Error Correction

While typing with a BCI, it could happen that the interface misclassifies and consequently mistypes the symbol intended by the user. As a result, for an ordinary BCI speller, one needs to foresee a “backspace” command for correction, or to use some brain potential connected to the subject's realization of an error (Error-related Potential) followed by some smart algorithm for correcting the mistyped character [118]. As yet another alternative, one might not perform any correction, but continue to type while relying on an incorporated language model that performs the correction automatically at a later stage.

The latter was explored in [103] for the P300 speller thereby assuming that for each spelled word the start and end points are determined correctly (*i.e.*, number of letters in typed word is correct). While typing each letter, BCI speller estimates probabilities of each letter to be intended by subject according to classifier outcomes, and rank them in descendent order according to these probabilities. When a whole word is spelled, a search through the attached dictionary is performed, and for each candidate word the sum of the above mentioned ranks of each letter in this word is computed. The word with the smallest sum of ranks is then selected as the mostly likely intended (“corrected”) word.

Other systems also allow for a correction of misspelled words to some extent. The word prediction module described in [114], which was discussed in the previous section, is based on the word prediction software WordQ2 [119], developed by Quillsoft Ltd. [120]. This software allows, for example, for the wrongly typed word “PLOS” (while the user intended to type “PLEASE”) to be included as the word “PLEASE” in the list of suggestions, hence, enabling the user to correct errors when using predictive spelling module technology [114].

### 3.6. Incorporation of Character Prediction Statistic into Classifier

It could also be possible to fuse the classifier with some natural language model. For example, assume one has typed the segment “WH” (the beginning of the word “WHAT”) and the next letter detected by the classifier is “T”. In this case it is not wise to present such a letter to the user since English does not have any word starting with “WHT”. Since it is clearly a mistake, it is better to use knowledge of what is possible and what not in a given language directly at the level of the classifier.

One can use an *n*-gram characters model for assessing, using the attached corpus, the probability of each possible character typed by taking into account the previously typed segment of length *n*−1 characters. Considering the previous example with “WH” and an 3-gram model, the system scans the corpus and counts all occurrences of “WHA”, “WHB”, “WHC”, … , “WHZ”, “WH ”, “WH.”, … After that, the probability of having as the next letter an “A” is estimated as the number of occurrences of “WHA” compared to the sum of all mentioned triples starting with “WH”. Such probabilities could be incorporated into the classifier by using, for example, a Bayesian interference strategy [44,121–123], thus for “correcting” the classifier output.

## 4. Assessment of BCI Spellers Based on Language Models

When assessing the benefits of incorporating additional technology into a BCI system, such as the ones based on language models, it is important to use some measure for characterizing the performance gain. Atraditional measure such as typing accuracy is not adequate, as it does not provide any information about the spelling speed, which is an important usability characteristic. The *information transfer rate* (ITR), proposed in [2], takes into account the accuracy, the number of possible selectable commands the interface supports, and the time required for communicating one command (one interface selection). But the ITR leads to ambiguities for some speller interfaces, such as for the one proposed in [90] (shown in Figure5). In such an interface, the number of possible commands could be either five (since five SSVEP stimuli are used for letter selection by moving the cursor left, right, up and down and for validating the selection) or 32 (if each character is considered to be selectable) [124]. Moreover, if one types text with a BCI, it is sometimes required to use “backspace” for correction. While using additional commands, as “backspace”, is seen as undesirable, the correct selection of the “backspace” command will increase the ITR of the assessed system, as pointed out in [125]. In addition, if one wants to compare character-by-character typing with a word completion strategy, a new problem arises. Incharacter-by-character typing four selections corresponds to maximally a four-letter word (if no mistakes occur), but the same four selections in a word completion strategy could result, for example, in a ten-letter word when, after spelling the first three letters, on a character-by-character basis, the fourth selection was used for choosing a ten-letter word from the list of suggestions. The ITR will treat the two cases in the same way, while it is clear that the latter one is much more beneficial. As a remedy, one could use the time spent on spelling some text [98]. However, in general, in different studies the spelled texts are usually different, and therefore we cannot use this time-based measure to compare different BCI-spellers.

Ryan and colleagues proposed in [114] to use the *output character per minute* (OCM) measure, which is estimated by taking the ratio of the total number of characters in the final text to the total time spent on spelling this text. They showed that while the standard ITR indicates a decrease from 19.39 ± 5.37 bit/min to 17.71 ± 5.38 bit/min by switching from character-by-character to predictive spelling, the OCM measure is more appropriate and characterizes the benefit obtained by incorporating a language model by an increase from 3.71 ± 0.75 character/min for character-by-character mode to 3.76 ± 0.75 character/min for predictive spelling.

Another strategy to overcome the limitation of the standard ITR measure in the case of text spelling was proposed by Kaufmann and colleagues [115]. Instead of estimating the ITR in terms of selections per minute, they suggested to estimate the true bit rate in terms of the communicated characters per time unit. They showed that in their experiments the standard ITR was only slightly better for predictive spelling (15.7 ± 5.7 bit/min) compared to character-by-character spelling (15.1 ± 5.6 bit/min), while the true ITR better characterized the benefits of the language model by producing 20.6 ± 5.3 bit/min for predictive spelling and 12.0 ± 2.7 bit/min character-by-character spelling.

So far there is no consensus what measure to use. Different studies exploit different techniques for performance assessment. In Table 1 we list the performance data of the reviewed studies, where the results with and without natural language models are indicated. Since the performance of a BCI-speller depends on several components (e.g., classifier, preprocessing and so on, see Section 2), we wanted to show only the effect of the language model while the other system components (like classifier, signal processing, …) remain the same.

## 5. Discussion

As is seen from Table 1, incorporating language models into BCI-spellers provides benefits in performance. In this way, language models could be seen as another way to improve the performance, in addition to a better classifier, more advanced signal processing, and so on. While the latter conventional methods are intended to change one of the blocks in Figure 1, the incorporation of a language model could be seen, in the majority of cases (for Sections 3.2–3.6), as an additional block in the scheme, which could also influence the classification step, its outcome, or the interface (display layout) itself.

Studies done so far with language models in BCI spellers only had a small amount of words/characters typed (less than 60 characters). Therefore, it is difficult to draw any solid and unbiased conclusions about the benefits of language models during a prolonged use of the system. However, one could expect that in this case the user could become more familiarized with the interface and its abilities and caveats.

Moreover, some interfaces [113,114] allow for the inclusion of the user's most frequent words and phrases, collected when using the BCI system, which could also speed up typing, especially in a word completion mode. On the other hand, with some of the language model implementations, as in [114], which were reported to increase the mental workload, the performance could even decrease after prolonged use. All these points indicate the necessity to perform longitudinal studies to properly evaluate the benefits of such implementations.

Even to date the potential benefits (if any) of some language models are not yet fully investigated. For example, the Dasher interface (see Section 3.2) is merely presented as a proof of concept for BCI. Whether it is beneficial or not still remains an open question. Additionally, some evaluations and comparisons were only done with simulated BCI spellers [99,107] or with off-line data [44,121]. All this still calls for on-line assessments of the proposed methodologies.

While the primary goal of BCI is to help patients suffering from locked-in syndrome, severe speech or motor disabilities, all studies with language models done so far only considered healthy subjects. It could very well be that some of the suggested methodologies, such as word completion, which require an increased cognitive load [114], are in fact infeasible for certain patient groups.

Another challenge is the design of an appropriate interface, tailored to the user. This could even start with the selection of the corpora so that the interface is better tuned to the user's language or his/her language capabilities. Human-machine interaction studies in this direction are needed. It would also be beneficial to have interfaces that work without requiring the user to switch between different interface layouts as, for example, in [112]. Such modifications could result in faster typing, since no commands for switching between interfaces are required. From the reviewed publications it is already seen that, for example, the list of word suggestions (during predictive spelling), integrated directly into interface, can reduce the mental workload [115]. All these could inspire the design and implementation of an interface that complies to with the main goals of AAL: to render the resulting system easily usable by the targeted user and not to increase its mental effort.

Most of the publications to language models in BCI spellers explore mainly only one of the language models presented in Section 3, while it is desirable to use several of them simultaneously to boost the performance. For example, a classifier for typing consecutive letters that takes into account letter probabilities depending on previously typed text (Section 3.6), could be easily connected to predictive word spelling (Section 3.4). The same principle of implementing several applications, according to the particular needs of AAL users, can be followed when integrating BCI spellers in AAL applications (e.g., in areas of safety, social environment, housework).

While the previous remarks are somewhat general, each method has its limitations and possible directions for improvement. For example, in [112] the word prediction model was supported by a dictionary containing the 49,142 most common words in English. This dictionary consisted of (mainly) the singular form of those words, whereas the user sometimes wanted to type words in plural. Since the word completion strategy used in this study had each word completed by adding a space after it, many users preferred the character-by-character spelling mode over the word completion one, since the latter required frequent corrections, by using the “backspace” command, and a retyping to obtain the plural form. As another example, we mention the word correction strategy proposed in [103] which can work under the following conditions: (a) the spelling system must exactly know where the intended word starts and ends (thus, a misspelled word-separating symbol could be considered as a part of the intended word); (b) the words can only contain letters (no digits and/or other characters); and (c) words not from the system dictionary are not supposed to be spelled. If at least one of these conditions is violated, then the described word correction strategy will be useless and will lead to a wrong textual output. Hence, further research is needed to overcome these limitations.

The potential benefits of BCI have been exploited in different BCI systems [68] including AAL applications. Particularly, BCI-spellers are in a position to improve the quality of life of people with particular communication needs [6] as is the case in the AAL community. Additionally, the various implementations of language models (e.g., completion, design of appropriate interfaces, avoid the switching between interfaces layouts, predictive characters selection) on BCI-spelling systems, as described in this review, could offer new ways to interact with assistive living, communication and control systems. Such implementations could support an active social environment in the context of rehabilitation [4], and AAL applications such as control environment and context awareness [8].

## 6. Conclusions

In this study we reviewed several approaches to boost the performance of existing BCI spellers by using language models. We categorized them based on the language model used and the way it is integrated. Different methods for assessing and comparing the performance of BCI spellers were discussed and adaptations to better account for the integrated language models suggested. We conclude that as a result of application of these language models, a significant improvement in spelling performance can be achieved, and new avenues of BCI integration in the AAL community charted such as social and environment control and rehabilitation.

## Figures and Tables

**Table 1.** Difference in performance of spelling interfaces with and without natural language models. The third column refers to the sections where they are discussed.

**Study**	**BCI Paradigm**	**Section Describing Model**	**Number of Subjects**	**Amount of Words/Characters Typed**	**Performance without Natural Language Model**	**Performance with Natural Language Model**
Kaufmann *et al.* 2012 [115]	ERP-based	3.4.	19	9 words/45 characters	15.1 ± 5.6 bit/min (ITR) 12.0 ± 2.7 bit/min (true ITR)	15.7 ± 5.7 bit/min (ITR) 20.6 ± 5.3 bit//min(true ITR)
Ryan *et al.* 2010 [114]	ERP-based	3.4.	24	Sentence with 58 characters	19.39 ± 5.37 bit/min (ITR) 3.71 ± 0.75 char/min (OCM)	17.71 ± 5.38 bit/min (ITR) 3.76 ± 0.75 char/min (OCM)
Volosyak *et al.* 2011 [112]	SSVEP	3.4.	7	Three phrases with in total 34 characters	29.98 ± 5.79 bit/min (true ITR)	32.71 ± 9.18 bit//min (true ITR)
Ahi *et al.* 2011 [103]	ERP-based	3.5.	14	10 words with 4 characters each	For 2 trials: 8.48 bit/min (ITR)	For 2 trials: 35.24 bit/min (ITR)
Ahi *et al.* 2011 [103]	ERP-based	3.5. + 3.1.	14	10 words with 4 characters each	For 2 trials: 8.48 bit/min (ITR)	For 2 trials: 55.32 bit/min (ITR)
Speier *et al.* 2012 [44]	ERP-based	3.6.	6	9 words with 5 letters each	22.07 ± 8.48 bit/min (ITR)	33.15 ± 12.37 bit/min (ITR)
D'Albis *et al.* 2012 [98]	MI	3.2.	3	Phrase with 20characters	12:56 min (spelling time)	10:38min (spelling time)
D'Albis *et al.* 2012 [98]	MI	3.4.	3	Phrase with 20 characters	12:56 min (spelling time)	6:27 min (spelling time)
D'Albis *et al.* 2012 [98]	MI	3.2. + 3.4.	3	Phrase with 20characters	12:56 min (spelling time)	6:09 min (spelling time)
Akram *et al.* 2013 [113]	ERP-based	3.4.	4	10 words	2.9 min (word spelling average time)	1.66 min (word spelling average time)
